# Development and validation of a preoperative prognostic index independent of TNM stage in resected non-small cell lung cancer

**DOI:** 10.1186/s12890-017-0529-9

**Published:** 2017-12-04

**Authors:** Shogo Kumagai, Satoshi Marumo, Machiko Arita, Keiji Yamanashi, Ryota Sumitomo, Yosuke Otake, Tsuyoshi Shoji, Motonari Fukui, Toshiro Katayama, Norihito Okumura, Cheng-Long Huang

**Affiliations:** 1 Respiratory Disease Center, Tazuke Kofukai Medical Research Institute, Kitano Hospital, 2-4-20 Ohgimachi, Kita-ku, Osaka, 530-8480 Japan; 2 Department of Respiratory Medicine, Ohara Memorial Kurashiki Central Hospital, Kurashiki, Japan; 3 Department of Thoracic Surgery, Ohtsu Red Cross Hospital, Otsu, Japan; 4 Faculty of Health Sciences, Department of Medical Engineering, Himeji Dokkyo University, Himeji, Japan; 5 Department of Thoracic Surgery, Ohara Memorial Kurashiki Central Hospital, Kurashiki, Japan

**Keywords:** Non-small cell lung cancer, Overall survival, Disease-free survival, Curative resection, Preoperative prognostic factors

## Abstract

**Background:**

Previously reported prognostic tools for patients with resected non-small cell lung cancer (NSCLC) include factors found postoperatively, but not preoperatively. However, it would be important to predict patient prognosis before NSCLC resection. To suggest a novel preoperative prognostic tool, we evaluated the relationship of preoperative prognostic factors with the survival of patients with resected NSCLC.

**Methods:**

We retrospectively reviewed the data of two independent cohorts of patients with completely resected NSCLC. To develop the prognostic index in one cohort, the overall survival (OS) was evaluated using the Cox proportional hazards model. We assessed the disease-free survival (DFS) and OS of three risk groups defined according to the prognostic index. Then, the prognostic index was validated in the other cohort.

**Results:**

Seven independent risk factors for OS were selected: age ≥ 70 years, ever-smokers, vital capacity <80%, neutrophil-to-lymphocyte ratio ≥ 2.1, cytokeratin 19 fragment >normal limit, non-usual interstitial pneumonia (UIP) pattern, and UIP pattern. Three risk groups were defined: low-risk (36.9%), intermediate-risk (54.0%), and high-risk (9.1%). In the derivation cohort, the 5-year DFS rate was 77.8%, 58.8%, and 22.6% (*P* < 0.001), and the 5-year OS rate was 95.2%, 70.4%, and 28.9% (*P* < 0.001), respectively. Multivariate analyses showed that the prognostic index predicted DFS and OS, independent of pathological stage and tumor histology, in both derivation and validation cohorts.

**Conclusions:**

We developed and validated a simple preoperative prognostic index composed of seven variables, which may help clinicians predict prognosis before surgery in patients with NSCLC.

**Electronic supplementary material:**

The online version of this article (10.1186/s12890-017-0529-9) contains supplementary material, which is available to authorized users.

## Background

Lung cancer is one of the most prevalent cancers and the leading cause of cancer death [1]. Pathological stage has been considered a strong prognostic factor for non-small cell lung cancer (NSCLC) [2]. However, it is known that outcomes of patients with the same pathological stage NSCLC are heterogeneous, with some experiencing early recurrences, while others remain alive without necessity for treatment. It is important to devise a prognostic index composed of preoperative factors complementary to the pathological stage, which could stratify the prognosis of NSCLC patients undergoing surgical treatment.

Several preoperative factors including blood test markers and high-resolution computed tomography (HRCT) findings have also been reported to predict the prognosis of patients with NSCLC. Of blood test markers, neutrophil-to-lymphocyte ratio (NLR), carcinoembryonic antigen (CEA) levels, and cytokeratin 19 fragment (CYFRA 21-1) levels have been demonstrated to predict the prognosis of patients with NSCLC [3–12]. In addition, previous reports have shown that interstitial lung disease (ILD) is associated with a poor prognosis of NSCLC [13–15].

There have been some reports about prognostic tools in resected NSCLC patients [16–20], including factors found after surgery, such as pathological tumor diameter and pathological lymph node status, but few prognostic tools composed only of preoperative factors have been developed. A prognostic index composed of preoperative prognostic factors complementary to the pathological stage may be important in the prediction of disease-free survival (DFS) and overall survival (OS) rates of NSCLC patients and in surgical decision-making. The aim of the present study was to identify preoperative prognostic factors associated with OS of resected NSCLC patients and to develop and validate a new prognostic index that could stratify the prognosis of NSCLC.

## Methods

This retrospective study aimed to identify preoperative prognostic factors and to develop and validate a new prognostic index of OS in patients with resected NSCLC.

### Patients

Patients diagnosed with NSCLC who received surgical treatment at Ohara Memorial Kurashiki Central Hospital between January 2007 and December 2011 (cohort 1) and the Tazuke Kofukai Medical Research Institute, Kitano Hospital between January 2007 and December 2012 (cohort 2) were retrospectively reviewed. All patients met the following criteria: pathological confirmation of NSCLC; no preoperative treatment; complete resections, more radical than or equal to segmentectomy with lymph node dissections or samplings; no microscopic residual tumor; no evidence of active infection such as pneumonia prior to surgery; evaluation of ILD with a HRCT scan, which was performed at diagnosis of lung cancer and was available for review; no history of acute exacerbation of chronic obstructive pulmonary disease or ILD within a month prior to surgery; and availability of laboratory data, including NLR, CEA and CYFRA 21-1, and follow-up information. Chronic obstructive pulmonary disease was diagnosed on the basis of the Global Initiative for Chronic Obstructive Lung Disease [21]. ILD was diagnosed only by two pulmonologists, based on medical history, physical examination, and abnormalities compatible with bilateral lung fibrosis on HRCT, according to the guidelines of the American Thoracic Society in 2011 [22]. The cases were categorized into two groups according to their radiologic appearance on HRCT: (1) usual interstitial pneumonia (UIP) pattern: characterized by the presence of basal-dominant reticular opacities and predominantly basal and subpleural distribution of honeycomb lesions, with multiple equal-sized cystic lesions of 2 to 10 mm diameter with a thick wall; and (2) non-UIP pattern: characterized by the presence of basal-predominant ground glass opacities and infiltrative shadows inconsistent with UIP patterns. We classified histology of lung cancer according to the World Health Organization guidelines [23]. Lung cancer pathological stages were based on the seventh edition of TNM classification of malignant tumors [2]. The study protocol was approved by the ethical committee of the Tazuke Kofukai Medical Research Institute, Kitano Hospital and Ohara Memorial Kurashiki Central Hospital in accordance with the Declaration of Helsinki.

### Evaluation of clinicopathological factors

The following clinical characteristics were reviewed from the available clinical records: age, sex, smoking history, resected side, surgical procedure, pathological stage, pathological tumor status, pathological lymph node status, histology of lung cancer, neutrophil counts, lymphocyte counts, NLR, CEA levels, CYFRA 21-1 levels, HRCT findings, percent vital capacity (%VC), percent forced expiratory volume in the first second (FEV1%), and causes of death. DFS was measured from the date of surgery until the date of recurrence or death, or until the date the patient was last known to be disease free. OS was measured from the date of surgery until the date of death from any cause or until the date on which the patient was last known to be alive.

### Statistical analysis

We estimated DFS and OS employing the Kaplan–Meier analysis [24]. Differences between survival curves were tested for statistical significance using the two-tailed log-rank test. We used the method of Holm to account for multiple testing [25]. Univariate and multivariate prognostic analyses were performed for DFS and OS outcomes using the Cox proportional hazards model. To devise a prognostic index, a multivariate proportional hazards model was employed to derive a final model of the variables that had a significant independent relationship with survival in cohort 1. We classified patients into the three risk groups: low-risk (5-year OS rate ≥ 80%), intermediate-risk (50% ≤5-year OS rate < 80%), and low-risk (5-year OS rate < 50%). Then, the devised prognostic index was validated in the data set of cohort 2. Receiver operating characteristic (ROC) curve analysis was used to determine the optimal cut-off values for NLR level, devised prognostic index, pathological stage; values with maximum joint sensitivity and specificity were selected. The DeLong’s test was used for comparison of the areas under the receiver operating characteristic curve (AUC). All statistical analyses were performed using R version 2.13.1 statistical software (R Foundation for Statistical Computing, Vienna, Austria). All *P* values are two-sided, and *P* values less than 0.05 were considered statistically significant.

## Results

### Patients

A total of 604 patients in cohort 1 and 333 patients in cohort 2 were enrolled in this study. The clinicopathological characteristics of patients are shown in Table 1. Significant differences between the two cohorts were found in sex, FEV1%, %VC, surgical procedures, clinical stage, clinical N factor, pathological T factor, pathological N factor, ILD, and postoperative chemotherapy. The median follow-up duration was 57.0 months (range: 0.20–99.3 months) in cohort 1 and 48.3 months (range: 0.9–103.7 months) in cohort 2. The 5-year DFS rate and OS rate of patients in cohort 1 were 63.2% (pathological stage I, 72.1%; pathological stage II, 40.8%; pathological stage IIIA, 28.3%) and 76.6% (pathological stage I, 81.5%; pathological stage II, 63.3%; pathological stage IIIA, 58.1%), respectively. The 5-year DFS rate and OS rate of patients in cohort 2 were 64.6% (pathological stage I, 76.4%; pathological stage II, 39.9%; pathological stage IIIA, 30.2%) and 76.6% (pathological stage I, 84.6%; pathological stage II, 63.5%; pathological stage IIIA, 49.4%), respectively.

**Table 1:** Clinicopathological characteristics of patients

Variables	Cohort 1 (*N* = 604)	Cohort 2 (*N* = 333)	*P*-value
Age (median), years	70 (33–90)	68 (19–87)	0.057
Sex (male/female)	376(62.3%)/228(37.7%)	181(54.4%)/152(45.6%)	0.022
Smoking history (current/former/never)	176(29.1%)/215(35.6%)/ 213(35.3%)	92(27.6%)/104(31.2%)/ 137(41.1%)	0.188
FEV1%, %	72.1 (33.4–157.4)	74.1 (31.5–100.0)	0.031
%VC, %	107.7 (49.8–156.0)	103.8 (56.9–171.8)	0.004
Resected side (right/left)	359(59.4%)/245(40.6%)	137(41.1%)/196(58.9%)	0.890
Surgical procedures (Pneumo/Lob/Seg)	4(0.7%)/467(77.3%)/ 133(22.0%)	1(0.3%)/293(88.0%)/ 39(11.7%)	<0.001
Clinical stage (I/II/IIIA)	470(77.8%)/98(16.2%)/ 36(6.0%)	253(76.0%)/41(12.3%)/ 39(11.7%)	0.005
Clinical T factor (T1/T2/T3/T4)	377(62.4%)/200(33.1%)/ 24(4.0%)/3(0.5%)	200(60.1%)/110(33.0%)/ 16(4.8%)/7(2.1%)	0.137
Clinical N factor (N0/N1/N2)	517(85.6%)/58(9.6%)/ 29(4.8%)	282(84.7%)/17(5.1%)/ 34(10.2%)	0.001
Pathological stage (I/II/IIIA)	457(75.7%)/86(14.2%)/ 61(10.1%)	240(72.1%)/42(12.6%)/ 51(15.3%)	0.061
Pathological T factor (T1/T2/T3/T4)	351(58.1%)/228(37.7%)/ 24(4.0%)/1(0.2%)	192(57.7%)/117(35.1%)/ 16(4.8%)/8(2.4%)	0.009
Pathological N factors (N0/N1/N2)	489(81.0%)/58(9.6%)/ 57(9.4%)	267(80.2%)/21(6.3%)/ 45(13.5%)	0.049
Histology (Ad/Sq/others)	440(72.8%)/123(20.4%)/ 41(6.8%)	260(78.1%)/55(16.5%)/ 18(5.4%)	0.180
Neutrophil counts (mean ± SD), mm-3	3800 ± 1400	3800 ± 1700	0.595
Lymphocyte counts (mean ± SD), mm-3	1700 ± 600	1700 ± 600	0.716
NLR (mean ± SD)	2.5 ± 1.5	2.6 ± 1.8	0.526
CEA (mean ± SD), ng/ml	7.0 ± 23.9	6.7 ± 22.0	0.830
CYFRA 21-1 (mean ± SD), ng/ml	1.9 ± 2.3	1.9 ± 3.1	0.886
ILD (UIP/non-UIP pattern)	35(5.8%, 7/28)	44(13.2%, 9/35)	<0.001
Postoperative adjuvant chemotherapy	199(32.9%)	170(51.1%)	<0.001
Recurrences of lung cancer	125(20.7%)	84(25.2%)	0.119
Cause of death (lung cancer/others/ unknown)	65(10.8%)/75(12.4%)/ 11(1.8%)	38(11.4%)/28(8.4%)/ 6(1.8%)	0.306

Abbreviations: *SD*, standard deviation; *Pneumo*, pneumonectomy; *Lob*, lobectomy or bilobectomy; *Seg*, segmentectomy; *Ad*, adenocarcinoma; *Sq*, squamous cell carcinoma; *NLR*, neutrophil to lymphocyte ratio; *CEA*, carcinoembryonic antigen; *CYFRA 21-1*, cytokeratin 19 fragment; *ILD*, interstitial lung disease; *UIP*, usual interstitial pneumonia

### Optimal cut-off values for NLR

A ROC curve for NLR was employed to determine the cut-off value in the data set of cohort 1. The AUC for NLR was 0.63 (95% confidence interval [CI], 0.58–0.68) (Additional file 1: Figure S1). A NLR of 2.1 corresponded to the maximum joint sensitivity and specificity on the ROC curve (72.8% sensitivity and 51.7% specificity).

### Factors associated with NSCLC prognosis

Univariate analysis showed ten significant risk factors for the OS in cohort 1 (Table 2). The multivariate analysis identified seven prognostic factors in cohort 1: age ≥ 70 years, ever-smokers, NLR ≥2.1, CYFRA 21-1 above the normal limit, non-UIP, and UIP.

**Table 2:** Prognostic impacts on overall survival in NSCLC in cohort 1

Univariate analysis		HR	95% CI			*P*-value
Age	≥70	2.39	1.69	–	3.36	<0.001
Sex	Male	3.29	2.15	–	5.02	<0.001
Smoking history	Ever	4.74	2.90	–	7.76	0.005
FEV1%	<70%	1.62	1.17	–	2.22	0.003
%VC	<80%	3.77	2.30	–	6.19	<0.001
NLR	≥2.1	2.31	1.60	–	3.32	<0.001
CEA (ng/mL)	>normal limit	2.11	1.50	–	2.96	<0.001
CYFRA21-1 (ng/mL)	>normal limit	3.85	2.51	–	5.91	<0.001
ILD^a^	Non-UIP	5.22	3.21	–	8.49	<0.001
	UIP	10.3	4.53	–	23.5	<0.001
Multivariate analysis		HR	95% CI			*P*-value
Age	≥70	1.65	1.16	–	2.36	0.005
Sex	Male	0.91	0.49	–	1.69	0.773
Smoking history	Ever	3.25	1.59	–	6.61	0.001
FEV1%	<70%	1.40	0.99	–	1.97	0.055
%VC	<80%	2.12	1.25	–	3.60	0.005
NLR	≥2.1	1.82	1.25	–	2.66	0.002
CEA (ng/mL)	>normal limit	1.39	0.97	–	1.97	0.070
CYFRA21-1 (ng/mL)	>normal limit	1.76	1.11	–	2.80	0.017
ILD^a^	Non-UIP	3.54	2.08	–	6.01	<0.001
	UIP	7.33	3.09	–	17.4	<0.001

Abbreviations: *NSCLC*, non-small cell lung cancer; *HR*, hazard ratio; 95% CI, 95% confidence interval; *NLR*, neutrophil to lymphocyte ratio; *CEA*, carcinoembryonic antigen; *CYFRA 21-1*, cytokeratin 19 fragment; *ILD*, interstitial lung disease; *UIP*, usual interstitial pneumonia

^a^The without-ILD group served as a reference group

### Development of the preoperative prognostic index

To develop the prognostic index, we examined two prognostic indexes (prognostic index 1 and 2) composed of the seven independent factors identified in the multivariate analysis: age ≥ 70 years, ever-smokers, %VC <80%, NLR ≥2.1, CYFRA 21-1 above the normal limit, non-UIP, and UIP. In the prognostic index 1, we allocated four points for UIP, two points for ever-smokers and non-UIP, and one point for the other four factors according to the hazard ratios (HRs) found in the multivariate analysis, while in the prognostic index 2 we allocated two points for UIP and one point for the other six factors for the ease of calculation. The comparisons of the AUCs for the ROC curves revealed no significant differences between the prognostic indexes in cohort 1 (0.78 [95% CI, 0.74–0.82; the prognostic index 1] vs. 0.77 [95% CI, 0.73–0.81; the prognostic index 2]; *P* = 0.174, Additional file 2: Figure S2). Therefore, taking the ease of calculation in clinical practices into consideration, we adopted the prognostic index 2 (Additional file 3: Table S1). Five-year OS rates according to risk scores of the prognostic index are shown in Additional file 3: Table S2. The AUC for the prognostic index in cohort 2 was 0.76 (95% CI, 0.70–0.83).

### Prognostic impacts of risk scores of the prognostic index

Univariate Cox regression analyses showed that risk scores of the prognostic index, clinical T factor, clinical N factor were significantly associated with OS (risk scores of the prognostic index: HR 2.18; 95% CI, 1.92–2.48; *P* < 0.001, clinical T factor: HR 1.95; 95% CI, 1.58–2.41; *P* < 0.001, clinical N factor: HR 1.70; 95% CI, 1.33–2.18; *P* < 0.001; Additional file 3: Table S3). Multivariate cox regression analyses demonstrated that risk scores of the prognostic index were significantly related with OS (HR 2.03; 95% CI, 1.78–2.32; *P* < 0.001), independent of clinical T factor (HR 1.41; 95% CI, 1.10–1.80; *P* < 0.001), and clinical N factor (HR 1.54; 95% CI, 1.20–1.99; *P* < 0.001).

**Fig. 1:**
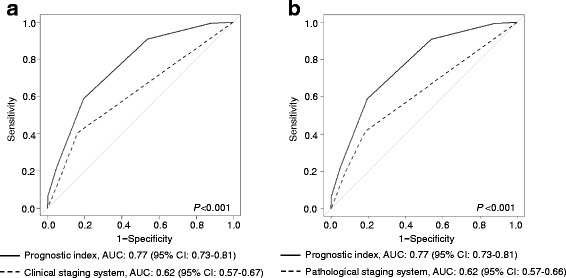
ROC curves for the prognostic index and pathological stage in cohort 1 (**a**) and cohort 2 (**b**) are shown

The AUC for risk scores of the prognostic index was 0.77 (95% CI, 0.73–0.81). The comparisons of the AUC in both cohorts revealed that the prognostic model presented a better diagnostic performance compared with the clinical staging system (0.77 vs. 0.62; *P* < 0.001; Fig. 1a), or with the pathological staging system (0.77 vs. 0.62; *P* < 0.001; Fig. 1b).

### Survival outcomes per prognostic index

**Fig. 2:**
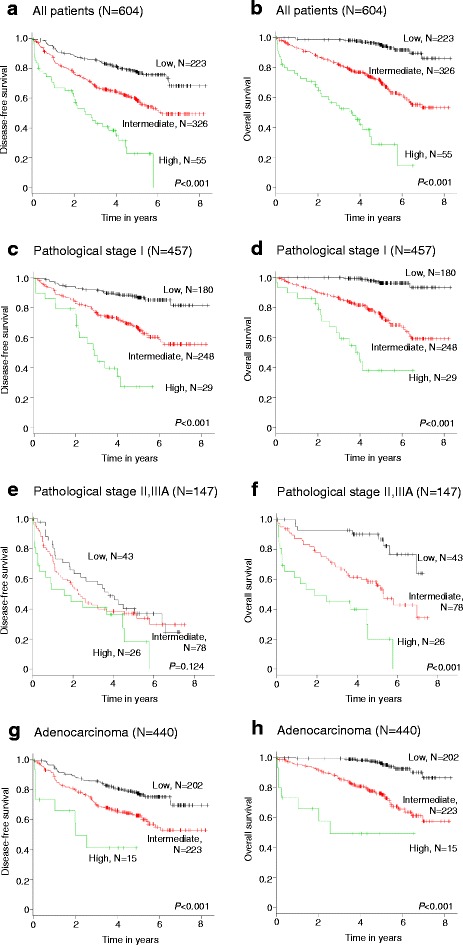
The Kaplan–Meier plots of DFS and OS in cohort 1 stratified by the prognostic index are shown (2A, 2B: DFS, OS in all patients, respectively) (2C, 2D: DFS, OS in patients with pathological stage I, respectively) (2E, 2F: DFS, OS in patients with pathological stage II, IIIA, respectively) (2G, 2H, DFS, OS in patients with adenocarcinoma, respectively)

DFS and OS were evaluated according to the three risk groups in cohort 1 with Kaplan–Meier analysis (Fig. 2). Patients were then stratified according to the following three risk groups: low-risk (no points or one point, 223 patients [36.9%]), intermediate-risk (two or three risk points, 326 patients [54.0%]), and high-risk (more than three points, 55 patients [9.1%]). In cohort 1, the 5-year DFS rate resulted in 77.8%, 58.8%, and 22.6% for patients at low risk, intermediate risk, and high risk, and the 5-year OS rate of 95.2%, 70.4%, and 28.9% in each risk category, respectively. In the analysis of DFS and OS of all patients included in cohort 1, significant differences in outcomes among the three groups were seen (DFS: *P* < 0.001; intermediate vs. low; *P* < 0.001, high vs. low; *P* < 0.001, high vs. intermediate; *P* < 0.001, Fig. 2a) (OS: *P* < 0.001; intermediate vs. low; *P* < 0.001, high vs. low; *P* < 0.001, high vs. intermediate; *P* < 0.001, Fig. 2B). In addition, subgroup analyses were performed (pathological stage I [DFS, Fig. 2c; OS, Fig. 2d], pathological stage II and IIIA [DFS, Fig. 2e; OS, Fig. 2f], and adenocarcinoma [DFS, Fig. 2g; OS, Fig. 2h]).

**Fig. 3:**
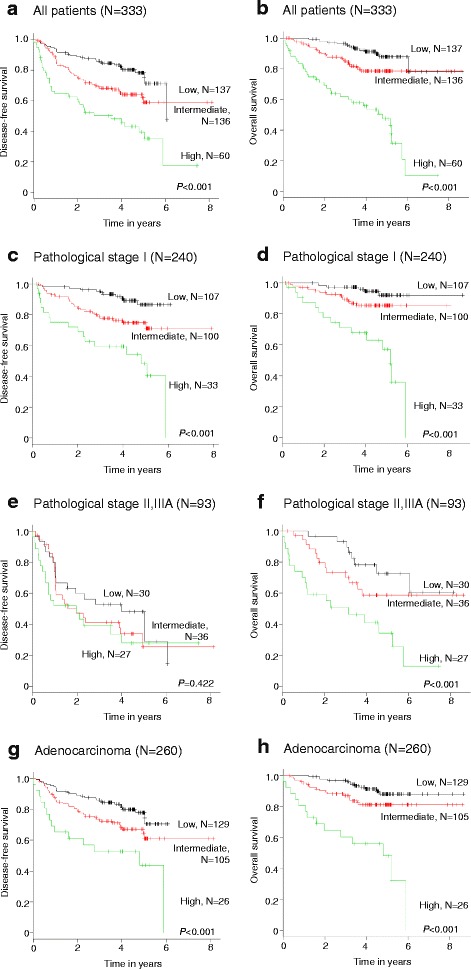
The Kaplan–Meier plots of DFS and OS in cohort 2 stratified by the prognostic index are shown (2A, 2B: DFS, OS in all patients, respectively) (2C, 2D: DFS, OS in patients with pathological stage I, respectively) (2E, 2F: DFS, OS in patients with pathological stage II, IIIA, respectively) (2G, 2H: DFS, OS in patients with adenocarcinoma, respectively)

Next, survivals were evaluated according to the three risk groups in cohort 2 (Fig. 3). In cohort 2, the 5-year DFS rate resulted in 78.3%, 61.8%, and 39.5% for patients at low risk (137 patients [41.1%]), intermediate risk (136 patients [40.8%]), and high risk (60 patients [18.0%]), and the 5-year OS rate of 87.8%, 78.4%, and 47.3% in each risk category, respectively. In the analysis of DFS and OS of all patients included in cohort 2, significant differences in outcomes among the three groups were seen (DFS: *P* < 0.001; intermediate vs. low; *P* = 0.005, high vs. low; *P* < 0.001, high vs. intermediate; *P* = 0.002, Fig. 3a) (OS: *P* < 0.001; intermediate vs. low; *P* = 0.015, high vs. low; *P* < 0.001, high vs. intermediate; *P* < 0.001, Fig. 3b). Subgroup analyses were performed, too (pathological stage I [DFS, Fig. 3c; OS, Fig. 3d], pathological stage II and IIIA [DFS, Fig. 3e; OS, Fig. 3f], and adenocarcinoma [DFS, Fig. 3g; OS, Fig. 3h]). All survival data, excluding DFS in pathological stage II and IIIA, were significantly stratified according to the three risk groups in both cohorts.

HRs of intermediate-risk group or high-risk compared to low-risk group were evaluated in cohort 1 (Additional file 3: Table S4) (DFS: intermediate vs. low; 2.13, 95% CI, 1.54–2.94; *P* < 0.001, high vs. low; 5.24, 95% CI, 3.41–8.03; *P* < 0.001, OS: intermediate vs. low; 6.20, 95% CI, 3.55–10.8; *P* < 0.001, high vs. low; 20.6, 95% CI, 10.9–38.7; *P* < 0.001). HRs adjusted for pathological stage and histology were evaluated in cohort 1 (DFS: intermediate vs. low; 2.04, 95% CI, 1.46–2.83; *P* < 0.001, high vs. low; 3.88, 95% CI, 2.38–6.34; *P* < 0.001, OS: intermediate vs. low; 5.69, 95% CI, 3.22–10.0; *P* < 0.001, high vs. low; 13.3, 95% CI, 6.68–26.4; *P* < 0.001).

In addition, HRs of intermediate-risk group or high-risk compared to low-risk group were also evaluated in cohort 2 (DFS: intermediate vs. low; 1.90, 95% CI, 1.19–3.02; *P* = 0.007, high vs. low; 3.90, 95% CI, 2.38–6.38; *P* < 0.001, OS: intermediate vs. low; 2.16, 95% CI, 1.13–4.13; *P* = 0.021, high vs. low; 7.71, 95% CI, 4.12–14.4; *P* < 0.001). HRs adjusted for pathological stage and histology were evaluated in cohort 2 (DFS: intermediate vs. low; 2.02, 95% CI, 1.26–3.24; *P* = 0.004, high vs. low; 4.92, 95% CI, 2.71–8.93; *P* < 0.001, OS: intermediate vs. low; 2.26, 95% CI, 1.16–4.41; *P* = 0.017, high vs. low; 11.3, 95% CI, 5.32–23.8; *P* < 0.001).

## Discussion

The present study demonstrated the association of age, ever-smokers, %VC, NLR, CYFRA 21-1 levels, and ILD with prognosis of NSCLC patients undergoing surgical treatment. Besides, the prognostic index of these factors could stratify well the prognosis of patients with NSCLC, even adjusted for pathological stage and histology, which was validated in an independent cohort. A previous study reported that age was an independent significant variable associated with survival of patients with NSCLC [26]. Some studies suggested that patients with NSCLC who were ever-smokers show a significantly worse prognosis than those who were never-smokers [27–31]. NLR is considered to reflect tumor-related inflammation and to predict prognosis of patients with NSCLC [3, 4]. We evaluated the association of CYFRA 21-1 levels and survival of patients with NSCLC. CYFRA 21-1, generally regarded as a marker of lung squamous cell carcinoma, is reported to have prognostic values in patients with NSCLC [9–12]. Although univariate analysis proposed significant prognostic values of CEA and CYFRA 21-1 levels, only CYFRA 21-1 levels were shown to be an independent prognostic factor in multivariate analysis. CEA levels have been known to be influenced by age or severity of ILD [32, 33]. which might suggest that CEA levels were not independent of age or ILD, in terms of the association with the survival of patients with NSCLC.

ILD have been known to be a negative prognostic factor of patients with resected NSCLC [13–15]. The multivariate analysis showed that the mortality HR of UIP pattern was twice higher than that of non-UIP pattern. Therefore, we allocated high points for UIP pattern in developing the prognostic index. Sato et al. reported that the five-year survivals of patients with stage IA tumor with %VC ≤ 80% were significantly lower than those with %VC >80% (20.0% vs. 64.3%) in resected NSCLC with a clinical diagnosis of ILD [34]. We demonstrated that %VC <80% was a significant independent unfavorable prognostic factor in patients with resected NSCLC. For patients with %VC <80%, the decision of pulmonary resection should be considered carefully.

Several studies have suggested tools predictive for the survival of patients with resected NSCLC [16–20]. There are some differences between our prognostic index and existing prognostic tools. First, our prognostic index is composed only of preoperatively available factors, while all the existing prognostic tools include factors found after surgery, such as pathological tumor diameter and pathological lymph node status. Therefore, our prognostic index could be used, auxiliary to the TNM staging system in deciding indications of surgery, or perhaps induction chemotherapy or chemoradiotherapy. Second, ILD, which was often overlooked or ignored in previous studies, was well evaluated in our study. The high-risk group patients in our study are either elderly, or they have poor lung function or ILD. When central tumors exist or when there is a suspicion of N1 disease, stereotactic radiotherapy is not an option and surgery is the only radical curative treatment option. The high-risk group patients are unlikely to be candidates for radical concurrent chemoradiotherapy. Hence, surgery is often their only radical curative treatment option for these patients. The choice of these patients could be between a higher risk operation and palliative treatment. Our prognostic index may be useful in identifying these patients, selecting the optimal treatment option and indeed in obtaining consent for high-risk treatment options, or allowing the patient to perhaps prefer a purely palliative or chemotherapy option. Third, our prognostic index is completely independent of TNM staging system. This would imply that our prognostic index would probably continue to be used in the time when the forthcoming (eighth) edition of the TNM classification for lung cancer supersedes the seventh edition of the TNM classification [35], while the existing predictive tools containing T or N factors of seventh edition will not. We believe that our prognostic index has additional values to the existing predictive tools.

This prognostic index also presented a good capacity of risk stratification of outcomes in subsets of pathological stage I and adenocarcinoma. In the subset of pathological stage II and IIIA, DFS was not clearly stratified, probably due to the small number of patients, although OS was well stratified in even a small number. This is possibly because patients with higher prognostic scores were less likely to enjoy the chance of receiving optimal treatment after recurrences. In the whole cohort, even adjusted for the pathological stage and histology, the impact of the prognostic index on OS and DFS was proven.

This study has several limitations. One major limitation is that the sample size of the present study was relatively small. Especially, the number of the patients with stage II, IIIA or those with histology except adenocarcinoma was small. The second is that the impact of the prognostic index was validated only in a separate single center cohort. For example, the proportion of patients with ILD in cohort 2 was larger than that in cohort 1. Indications for operations of NSCLC patients with ILD differ among institutions. Therefore, the results of this study should be validated in larger multicenter cohorts. The other is that it is unclear whether the prognostic index is applicable for estimation of the prognosis of patients with advanced NSCLC, because the prognostic index was devised in the analysis of resected NSCLC patients. Prospective larger studies are necessary to solve these limitations.

## Conclusions

A new prognostic index composed of the seven prognostic factors identified prior to surgery, which is easily calculated in the clinical practice, was proven to predict DFS and OS in patients with resected NSCLC in one cohort. In addition, this result was validated in a second, different cohort. This prognostic index may also be helpful in stratifying patients in prospective trials.

## Additional files

Additional file 1: Figure S1. The ROC curve of NLR is shown. (PDF 52 kb)

Additional file 2: Figure S2. The comparisons of the AUCs for the ROC curves between the prognostic index 1 and 2 are shown. (PDF 53 kb)

Additional file 3: Table S1. Prognostic index calculation of scores composed of independent risk factors is shown. **Table S2.** Five-year overall survivals according to each risk score are shown. Table S3. Impacts of risk scores on overall survival in NSCLC in cohort 1 are shown. Table S4. Relative risk according to risk groups as defined by the prognostic index are shown. (DOCX 23 kb)

## Abbreviations

%VC: percent vital capacity
AUC: areas under the receiver operating characteristic curve
CEA: carcinoembryonic antigen
CI: confidence interval
CYFRA 21-1: cytokeratin 19 fragment
DFS: disease-free survival
FEV1%: percent forced expiratory volume in the first second
HR: hazard ratio
HRCT: high-resolution computed tomography
ILD: interstitial lung disease
NLR: neutrophil-to-lymphocyte ratio
NSCLC: non-small cell lung cancer
OS: overall survival
ROC: Receiver operating characteristic
UIP: usual interstitial pneumonia
