# Supplementary figures and images for: Development and validation of a preoperative prognostic index independent of TNM stage in resected non-small cell lung cancer
Source: BMC Pulm Med. 2017 Dec 4;17:166. doi: 10.1186/s12890-017-0529-9 (PMC5715717; doi:10.1186/s12890-017-0529-9)

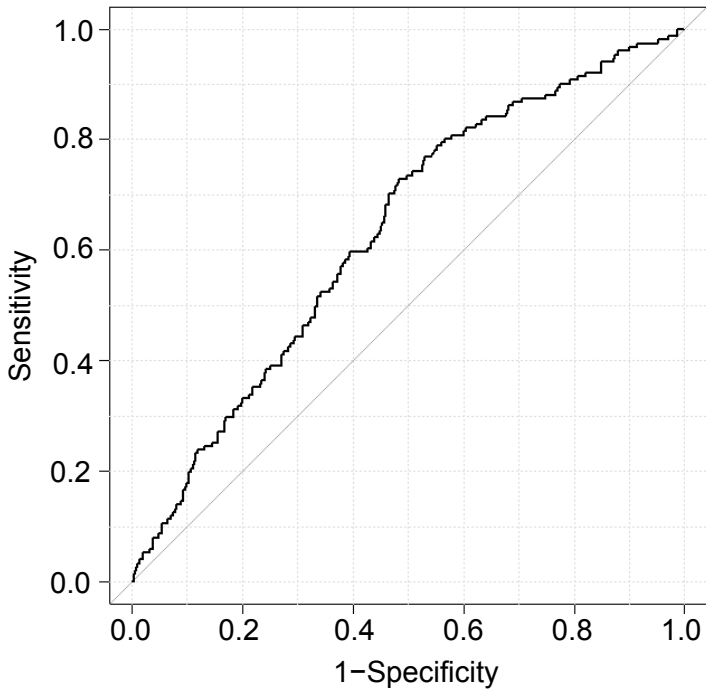

— NLR, AUC: 0.63 (95% CI: 0.58-0.68)

Supplement: Supplementary file 1 — The ROC curve of NLR is shown. (PDF 52 kb) [file 12890_2017_529_MOESM1_ESM.pdf]

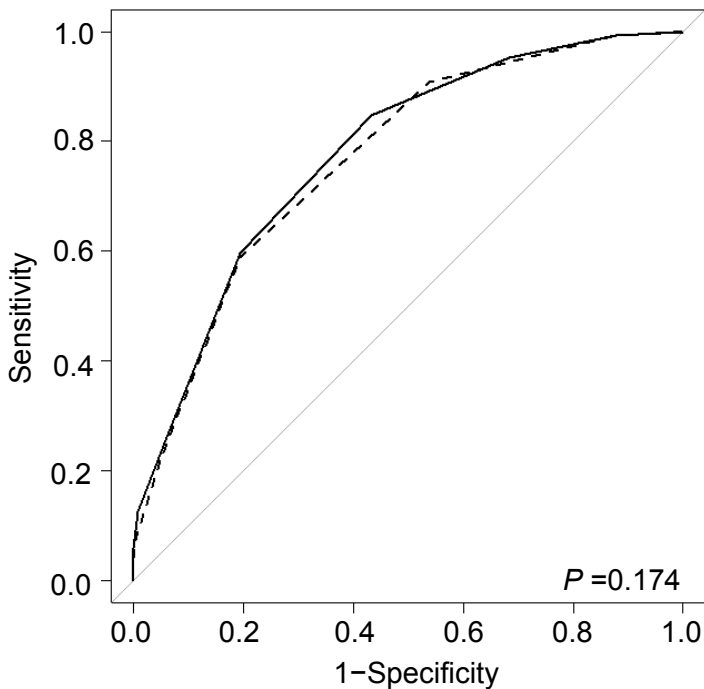

Supplement: Supplementary file 2 — The comparisons of the AUCs for the ROC curves between the prognostic index 1 and 2 are shown. (PDF 53 kb) [file 12890_2017_529_MOESM2_ESM.pdf]
