# Supplementary material for: Development and validation of a preoperative prognostic index independent of TNM stage in resected non-small cell lung cancer
Source: BMC Pulm Med. 2017 Dec 4;17:166. doi: 10.1186/s12890-017-0529-9 (PMC5715717; doi:10.1186/s12890-017-0529-9)
Supplement: Supplementary file 3 — Prognostic index calculation of scores composed of independent risk factors is shown. Table S2. Five-year overall survivals according to each risk score are shown. Table S3. Impacts of risk scores on overall survival in NSCLC in cohort 1 are shown. Table S4. Relative risk according to risk groups as defined by the prognostic index are shown. (DOCX 23 kb) [file 12890_2017_529_MOESM3_ESM.docx]

| Supplementary table 1. Prognostic index calculation | | |
| --- | --- | --- |
| Variables |  | scores |
| Age | ≥70 | 1 |
| Smoking history | Ever | 1 |
| %VC | <80% | 1 |
| NLR | ≥2.1 | 1 |
| CYFRA21-1 (ng/mL) | > normal limit | 1 |
| ILD* | Non-UIP | 1 |
|  | UIP | 2 |
| Risk groups | | Total scores |
| Low-risk group | | 0-1 |
| Intermediate-risk group | | 2-3 |
| High-risk group | | 4-7 |
| Abbreviations: NLR, neutrophil to lymphocyte ratio; CYFRA 21-1, cytokeratin 19 fragment; ILD, interstitial lung disease; UIP, usual interstitial pneumonia | | |

| Supplementary table 2. Five-year overall survivals according to each risk score | | | |
| --- | --- | --- | --- |
| Risk scores | N=604 | | 5-year OS rate |
| 0 | 56 | (9.3%) | 100.0% |
| 1 | 167 | (27.6%) | 93.6% |
| 2 | 204 | (33.8%) | 77.5% |
| 3 | 122 | (20.2%) | 58.5% |
| 4 | 44 | (7.3%) | 34.8% |
| 5 | 88 | (14.6%) | 16.7% |
| 6 | 2 | (0.3%) | 0.0% |
| 7 | 1 | (0.2%) | 0.0% |

| Supplementary table 3. Impacts of risk scores on overall survival in NSCLC in cohort 1 | | | | | |
| --- | --- | --- | --- | --- | --- |
| Univariate analysis | HR | 95% CI | | | *P*-value |
| Risk scores of prognostic index | 2.18 | 1.92 | − | 2.48 | <0.001 |
| Clinical T factor | 1.95 | 1.58 | − | 2.41 | <0.001 |
| Clinical N factor | 1.70 | 1.33 | − | 2.18 | <0.001 |
| Multivariate analysis | HR | 95% CI | | | *P*-value |
| Risk scores of prognostic index | 2.03 | 1.78 | − | 2.32 | <0.001 |
| Clinical T factor | 1.41 | 1.10 | − | 1.80 | <0.001 |
| Clinical N factor | 1.54 | 1.20 | − | 1.99 | <0.001 |
| Abbreviations: NSCLC, non-small cell lung cancer; HR, hazard ratio; 95% CI, 95% confidence interval; | | | | | |

| Supplementary table 4. Relative risk according to risk group as defined by the prognostic index | | | | | | | | | | |
| --- | --- | --- | --- | --- | --- | --- | --- | --- | --- | --- |
|  | HR | 95% CI | | | *P*-value | Adjusted HR* | 95% CI | | | *P*-value |
| DFS in cohort 1 |  |  |  |  |  |  |  |  |  |  |
| Low-risk group | ref |  |  |  |  | ref |  |  |  |  |
| Int-risk group | 2.13 | 1.54 | − | 2.94 | <0.001 | 2.04 | 1.46 | − | 2.83 | <0.001 |
| High-risk group | 5.24 | 3.41 | − | 8.03 | <0.001 | 3.88 | 2.38 | − | 6.34 | <0.001 |
| OS in cohort 1 |  |  | | |  |  |  | | |  |
| Low-risk group | ref |  |  |  |  | ref |  |  |  |  |
| Int-risk group | 6.20 | 3.55 | − | 10.8 | <0.001 | 5.69 | 3.22 | − | 10.0 | <0.001 |
| High-risk group | 20.6 | 10.9 | − | 38.7 | <0.001 | 13.3 | 6.68 | − | 26.4 | <0.001 |
| DFS in cohort 2 |  |  | | |  |  |  | | |  |
| Low-risk group | ref |  |  |  |  | ref |  |  |  |  |
| Int-risk group | 1.90 | 1.19 | − | 3.02 | 0.007 | 2.02 | 1.26 | − | 3.24 | 0.004 |
| High-risk group | 3.90 | 2.38 | − | 6.38 | <0.001 | 4.92 | 2.71 | − | 8.93 | <0.001 |
| OS in cohort 2 |  |  | | |  |  |  | | |  |
| Low-risk group | ref |  |  |  |  | ref |  |  |  |  |
| Int-risk group | 2.16 | 1.13 | − | 4.13 | 0.021 | 2.26 | 1.16 | − | 4.41 | 0.017 |
| High-risk group | 7.71 | 4.12 | − | 14.4 | <0.001 | 11.3 | 5.32 | − | 23.8 | <0.001 |
| Abbreviations: NSCLC, non-small cell lung cancer; HR, hazard ratio; 95% CI, 95% confidence interval; DFS, disease-free survival; OS, overall survival; Int, intermediate | | | | | | | | | | |
| *HR was adjusted for pathological stage, histology. | | | | | | | | | | |
